# Population pharmacokinetic and pharmacodynamic properties of artesunate in patients with artemisinin sensitive and resistant infections in Southern Myanmar

**DOI:** 10.1186/s12936-018-2278-5

**Published:** 2018-03-23

**Authors:** Jesmin Permala Lohy Das, Myat P. Kyaw, Myat H. Nyunt, Khin Chit, Kyin H. Aye, Moe M. Aye, Mats O. Karlsson, Martin Bergstrand, Joel Tarning

**Affiliations:** 1 Department of Pharmaceutical Biosciences, Uppsala University, Uppsala, Sweden; 2 Department of Medical Research, Yangon, Republic of the Union of Myanmar; 3 Mahidol-Oxford Tropical Medicine Research Unit, Faculty of Tropical Medicine, Mahidol University, Bangkok, Thailand; 4 Centre for Tropical Medicine, Nuffield Department of Clinical Medicine, University of Oxford, Oxford, UK

**Keywords:** Malaria, Resistance, Parasite clearance, Artemisinin, Pharmacokinetics, Pharmacodynamics, Nonlinear mixed-effects modelling

## Abstract

**Background:**

Artemisinins are the most effective anti-malarial drugs for uncomplicated and severe *Plasmodium falciparum* malaria. However, widespread artemisinin resistance in the Greater Mekong Region of Southeast Asia is threatening the possibility to control and eliminate malaria. This work aimed to evaluate the pharmacokinetic and pharmacodynamic properties of artesunate and its active metabolite, dihydroartemisinin, in patients with sensitive and resistant *falciparum* infections in Southern Myanmar. In addition, a simple nomogram previously developed to identify artemisinin resistant malaria infections was evaluated.

**Methods:**

Fifty-three (n = 53) patients were recruited and received daily oral artesunate monotherapy (4 mg/kg) for 7 days. Frequent artesunate and dihydroartemisinin plasma concentration measurements and parasite microscopy counts were obtained and evaluated using nonlinear mixed-effects modelling.

**Results:**

The absorption of artesunate was best characterized by a transit-compartment (n = 3) model, followed by one-compartment disposition models for artesunate and dihydroartemisinin. The drug-dependent parasite killing effect of dihydroartemisinin was described using an Emax function, with a mixture model discriminating between artemisinin sensitive and resistant parasites. Overall, 56% of the studied population was predicted to have resistant malaria infections. Application of the proposed nomogram to identify artemisinin-resistant malaria infections demonstrated an overall sensitivity of 90% compared to 55% with the traditional day-3 positivity test.

**Conclusion:**

The pharmacokinetic-pharmacodynamic properties of artesunate and dihydroartemisinin were well-characterized with a mixture model to differentiate between drug sensitive and resistant infections in these patients. More than half of all patients recruited in this study had artemisinin-resistant infections. The relatively high sensitivity of the proposed nomogram highlights its potential clinical usefulness.

## Background

Artemisinin-based combination therapy (ACT) is the recommended first-line therapy against uncomplicated *Plasmodium falciparum* malaria worldwide [1]. ACT is the most effective anti-malarial treatment today and consist of an artemisinin derivative and a partner drug [2]. The short-acting but potent artemisinin component eliminates the majority of parasites during the first 3 days of treatment and the slow acting and less potent partner drug removes residual parasites to prevent recrudescent infections. The introduction of ACT has contributed substantially to the reduction in malaria-related mortality and morbidity during the last decade [3].

However, the effectiveness of artemisinins is threatened in Southeast Asia due to widespread artemisinin resistance in the region [4]. Artemisinin resistant *P. falciparum* is defined clinically as delayed parasite clearance [5]. The clearance of microscopy detectable parasites by 24–48 h after the first dose of ACT is a typical indication of *P. falciparum* being fully susceptible to artemisinins [6], and parasite detection at 72 h after treatment initiation is interpreted as possible resistance [7]. Delayed parasite clearance results in higher parasite densities to be eliminated by the partner drug, causing an increased selection pressure for partner drug resistance [8]. High-grade resistance to both dihydroartemisinin and its partner drug, piperaquine, is now seen in Cambodia, Thailand, Laos and Vietnam resulting in unacceptably high clinical failure rates [9–11].

In early 2009, delayed parasite clearance was observed also in Myanmar [5, 12]. This was recently confirmed by the presence of slow parasite clearance as well as the in vivo molecular marker for artemisinin resistance (i.e. *kelch 13* mutations) [13, 14]. The fear is that this resistance genotype will spread through Bangladesh, India and find its way to the African continent similar to what was seen for chloroquine and sulfadoxine–pyrimethamine resistance [15].

Even though the discovery of *kelch 13* marker for artemisinin resistance has changed the resistance monitoring paradigm, the day-3 positivity tests is still being used clinically to detect resistant malaria infections due to advantageous of cost, easy accessibility, and field applicability. However, the performance of this test is deemed rather ineffective as the absolute parasite clearance time, and therefore also day-3 positivity, is very much dependent on the baseline parasite density [7]. The day-3 positivity test is particularly disadvantaged in high-endemic settings since acquired immunity often results in lower baseline parasitaemia and a faster parasite clearance rate. Thus, a new simple algorithm (baseline-adapted nomogram) which also takes into account the baseline density was proposed recently based on an analysis of data from a study in Thailand and Cambodia [4].

The primary aim of this study was to describe and evaluate the population pharmacokinetic and pharmacodynamic properties of artesunate (ARS) and its active metabolite, dihydroartemisinin (DHA), in the treatment of sensitive and resistant *P. falciparum* infections in Myanmar. A secondary aim was to perform an external validation of the recently proposed baseline-adapted nomogram to identify artemisinin resistant malaria infections.

## Methods

### Study design

This was a non-randomized, single arm, open-labelled clinical trial conducted in Palm Tree plantation site Hospital in Kawthaung in southern Myanmar. The trial was conducted to assess parasite clearance times in patients with uncomplicated *P. falciparum* malaria after ARS monotherapy. Clinical outcome and non-compartmental pharmacokinetic results have been published in full elsewhere (5). Ethical approval was obtained from the ethical review committees of the Department of Medical Research (Lower Myanmar), the Myanmar Ministry of Health, and the World Health Organization (WHO); Trial registration: Australian New Zealand Clinical Trials Registry ACTRN12610000896077.

Fifty-three (n = 53) patients who met all of the inclusion criteria and none of the exclusion criteria were recruited. Inclusion criteria; 18–55 years old, mono-infection with *P. falciparum*, asexual parasite density of 10,000–100,000/µl, fever in last 24 h, ability to tolerate oral intake of ARS, agreement to comply with the study protocol, and provision of written informed consent. Exclusion criteria; severe malaria, severe malnutrition, pregnancy, lactation, mixed malaria infection, clinical evidence of infection other than malaria, history of chronic medical illness, splenectomy, hypersensitivity to ARS or related compounds, or reported use of drugs with anti-malarial activity within 48 h before enrollment.

Study drug was procured from Guilin Pharmaceutical Co. Ltd. (Shanghai, China); lot Number AS091001. All patients received directly observed oral ARS monotherapy (4 mg/kg/day) once daily for 7 days, administered with 8 oz. of milk.

Venous blood samples were taken immediately before and at 0.25, 0.5, 0.75, 1.0, 1.25, 1.5, 3, 4, 6 and 8 h after the first dose. Within 15 min of collection, blood was centrifuged at 4 °C at 2000×*g* for 7 min and plasma was stored in liquid nitrogen until analysis. All samples were freighted on dry ice to the Department of Clinical Pharmacology at Mahidol-Oxford Tropical Medicine Research Unit (Bangkok, Thailand) where the plasma samples were analysed. The laboratory is a participant in the QA/QC proficiency testing programme supported by the Worldwide Antimalarial Resistance Network (WWARN) [16]. Plasma concentrations of ARS and DHA were measured by liquid chromatography-tandem mass spectroscopy using a published and validated method [17]. The observed total assay coefficients of variation was below < 8% in all quality control samples which were in concordance with FDA requirements, i.e. variations less than 15% at each low, medium and high concentration [18]. The lower limits of quantification (LLOQ) were 1.2 and 2.0 ng/ml for ARS and DHA, respectively. Microscopy parasite counts was performed every 12 h until two consecutive negative smears using Giemsa-stained thick and thin blood smears. Parasites were counted against 200 or 500 white blood cells and multiplied by an assumed white blood cell count of 6000/μl.

### Population pharmacokinetic-pharmacodynamic modelling

Data analysis was performed using nonlinear mixed-effects modelling implemented in the NONMEM software, v.7.3 (ICON Development Solutions, Ellicott City, MD) [19]. Xpose v.4.5.3 was used for graphical analysis and visual diagnostics of the model [20, 21]. Pearl-Speaks-NONMEM (PsN), v. 4.5.5 [22], Xpose, Pirana, v. 2.9.2 (21), and R, v. 3.2.4 (The R Foundation for Statistical Computing) [23, 24] were used for other post-processing, model diagnostics, graphical analysis and automation.

Model selection was guided by plausible parameter estimates, precision of parameters, visual diagnostics and minimum objective function value (OFV) computed by NONMEM as proportional to minus twice the log likelihood of the data. A drop in OFV of 3.84 or more was considered a significant improvement (p < 0.05) between two hierarchical models after inclusion of one additional parameter (one degree of freedom difference). Visual predictive checks (VPCs) were performed (2000 simulations) to evaluate the predictive performance of the pharmacokinetic and pharmacodynamic models. The reliability of individual parameter estimates and goodness-of-fit plots were assessed by evaluating eta and epsilon shrinkages. The 95% confidence intervals (CIs) of the estimated parameters and parameter uncertainties were calculated using the Sampling Importance Resampling (SIR) method [25, 26].

### Population pharmacokinetics

Observed concentrations of ARS and DHA (molar units) were transformed into their natural logarithms and modelled simultaneously. Complete metabolic in vivo conversion of ARS into DHA was assumed throughout modelling [27]. Observations falling below the LLOQ was included and analysed using the Laplacian estimation method (i.e. previously established likelihood based M3-method) [29–30]. Models with one, two and three disposition compartments were explored and the best performing disposition model was carried forward to evaluate different absorption models, i.e. first-order absorption with and without lag-time, zero-order absorption, sequential zero- and first-order absorption and transit-compartment absorption. The number of transit compartments was determined by stepwise addition of one to ten transit compartments to minimize the OFV. The drug transfer rate between transit compartments (K_TR_) was described by the equation below:

1 $$\varvec{K_{TR}} = \frac{{\left( {\varvec{n} + {\mathbf{1}}} \right)}}{{\varvec{MTT}}}$$

where MTT is the mean transit time and n is the number of transit compartment.

Between subject variability (BSV) was modelled exponentially as described below:

2 $$\varvec{\theta}_{\varvec{i}} = \varvec{\theta}_{{\varvec{TV}}} \times \varvec{e}^{{\upeta_{\varvec{i}} }}$$

where $\varvec{\theta}_{\varvec{i}}$ is the estimated individual parameter, $\varvec{\theta}_{{\varvec{TV}}}$ is the estimated population parameter value, and $\varvec{e}^{{\upeta_{\varvec{i}} }}$ represents the BSV, assumed to be independent and normally distributed around zero with a variance ω^2^. The unexplained residual variability (RUV) was estimated by separate additive error models for log-transformed ARS and DHA concentrations (i.e. equal to exponential error models on an arithmetic scale).

Clearance and volume of distribution of both parent and metabolite were scaled allometrically using body weight. Scaled body weight was raised to the power of 0.75 and 1 for clearance and volume parameters, respectively, and centered on the median weight of the population.

Due to lack of subsequent concentration measurements after first dose, malaria disease effect as a covariate on the absorption rate (MTT) and relative bioavailability (F) was implemented a priori based on a previous analysis in a neighbouring region (Thailand–Cambodia) according to the equations below (i.e. Eq. 3 for MTT and Eq. 4 for F).

3 $$\varvec{\theta}_{\varvec{i}} =\varvec{\theta}_{{\varvec{TV}}} \times \left( {{\mathbf{1}} + \varvec{PARA}_{{\varvec{MTT}}} \times \left( {{\mathbf{Log}}\left( {\varvec{PARA}_{\varvec{i}} } \right) - {\mathbf{Log}}\left( {\varvec{PARA}_{{{\mathbf{min}}}} } \right))} \right)} \right.$$

4 $$\varvec{\theta}_{\varvec{i}} =\varvec{\theta}_{{\varvec{TV}}} \times \left( {{\mathbf{1}} + \left[ {\frac{{\left( {{\mathbf{Log}}\left( {\varvec{PARA}_{\varvec{i}} } \right) - {\mathbf{Log}}\left( {\varvec{PARA}_{{{\mathbf{min}}}} } \right)) \times \varvec{PARA}{\mathbf{max}}_{\varvec{F}} } \right. }}{{\left( {{\mathbf{Log}}\left( {\varvec{PARA}_{\varvec{i}} } \right) - {\mathbf{Log}}\left( {\varvec{PARA}_{{{\mathbf{min}}}} } \right)} \right) + \left( {\varvec{PARA}{\mathbf{50}}_{\varvec{F}} - {\mathbf{Log}}\left( {\varvec{PARA}_{{{\mathbf{min}}}} } \right)} \right)}} } \right]} \right)$$

where PARA_MTT_ is the estimated linear effect of parasite density on MTT, PARAmax_F_ is the maximum effect of parasite density on F, PARA50_F_ is the parasite density which produces 50% of the maximum covariate response, and PARA_i_ is the individual parasite density. All other potentially influential covariates (i.e. age, sex, baseline hemoglobin, and temperature) were evaluated on all parameters using a stepwise forward inclusion (p < 0.05) and backward deletion (p < 0.01) covariate modelling approach. Individual pharmacokinetic parameter estimates from the final pharmacokinetic model were imputed as posterior Bayes estimates into the pharmacokinetic-pharmacodynamic model.

### Population pharmacodynamics

Total circulating parasite biomass was calculated by multiplying parasite counts by the individual patient’s estimated blood volume, computed using Nadler’s formula which takes into account gender, body weight and height [31]. Total parasite densities were transformed into their natural logarithms and modelled initially using a simplified one-compartment parasite model [32]. Data below the LLOQ (less than 12 parasites per µl) were modelled with the M3-method [28, 30]. Parasites were assumed to have a tenfold multiplication per asexual cycle of 48 h [33]. Drug effects were evaluated using a direct response model (i.e. basic Emax model and sigmoidal Emax model) and an indirect response model (i.e. delayed effect model/hysteresis). Individually predicted plasma concentrations of DHA were used to evaluate the drug-dependent killing of parasites (KILL), leading to an approximate log-linear reduction in parasite numbers with time (Eq. 5).

5 $$\varvec{KILL} = \frac{{\varvec{E}_{{{\mathbf{max}}}} \times \varvec{Ce}}}{{\varvec{EC}_{{{\mathbf{50}}}} + \varvec{Ce}}}$$

where Emax is the maximum parasite killing effect, *Ce* is the DHA drug concentration in the effect compartment and EC_50_ is the concentration which produces 50% of maximum killing effect. A mixture model to identify artemisinin resistant and artemisinin sensitive parasite infections was implemented on Emax, and the probability of having an artemisinin resistant infection (**MIXi** = 2) was estimated on a logit-transformed domain (Eq. 6).

6 $${\mathbf{log}}\,{\mathbf{it}}\left[ {{\mathbf{P}}\left( {{\mathbf{MIXi}} = {\mathbf{2}}} \right)} \right] = {\mathbf{ln}}\left( {\frac{{{\varvec{\uptheta}}_{{{\mathbf{PMIX2}}_{{{\mathbf{resistant}}}} }} }}{{{\mathbf{1}} - {\varvec{\uptheta}}_{{{\mathbf{PMIX2}}_{{{\mathbf{resistant}}}} }} }}} \right)$$

where ${\varvec{\uptheta}}_{{{\mathbf{PMIX2}}_{{{\mathbf{resistant}}}} }}$ is the population probability of belonging to mixture 2 (artemisinin resistant infection). Individual Emax-values associated with an artemisinin sensitive infection (higher parasite killing effect) and artemisinin resistant infection were parameterized as below to improve stability, and to constrain all individual Emax_Si_ values to be greater than the typical population Emax value associated with a resistant infection [34].

7 $$\varvec{E}{\mathbf{max}}_{{\varvec{Si}}} =\varvec{\theta}_{{\varvec{TVR}}} + \left( {\varvec{\theta}_{{\varvec{TVS}}} -\varvec{\theta}_{{\varvec{TVR}}} } \right) \times \varvec{e}^{{\upeta_{\varvec{S}} }}$$

8 $$\varvec{E}{\mathbf{max}}_{{\varvec{Ri}}} =\varvec{\theta}_{{\varvec{TVR}}} \times \varvec{e}^{{\upeta_{\varvec{R}} }}$$

where **θ**_**TVR**,_ and **θ**_**TVS**_ are the estimated typical parameter values for resistant infections and sensitive infections, respectively, with their variance (η). A frequentist prior functionality [35] was implemented a priori to support the estimation of EC_50_ and Emax in patients with artemisinin sensitive infections. The EC_50_ and Emax estimates from the artemisinin sensitive subgroup in a previous analysis of patients from Thailand and Cambodia (EC_50_: 34.9 nM [31.7% RSE] and Emax: 0.273 h^**−**1^ [6.25% RSE]) were used for this purpose. This was based on the assumption that the drug sensitivity in the non-resistant population is likely to be similar in the greater Mekong sub region.

### Evaluation of the resistance nomogram

A simple diagnostic method to detect resistant malaria infections was recently developed, based on data from Thailand and Cambodia [36]. This new diagnostic method demonstrated to be a promising alternative to the current practice of day-3 positivity test as a proxy of having a drug resistant malaria infection [36]. The developed nomogram used baseline parasite counts to select the time point post-treatment to assess the parasite count. These post-treatment parasite counts in relation to baseline parasite density were used to identify patients with resistant infections.

The nomogram was applied in this current dataset to act as external validation of the proposed method. The individual parasite ratio (*Ratio*_*i*_) of baseline parasite count and that at the chosen assessment time point j (i.e. 24, 48 or 72 h post-treatment) was calculated as below (Eq. 9)

9 $$Ratio_{{i,j\left( {24,48,72} \right)}} = \text{Log}\left( {PARA} \right)_{i,0} - \text{Log}\left( {PARA} \right)_{{i,j\left( {24,48,72} \right)}}$$

The nomogram-suggested “cut-off” value for chosen time points (i.e. 1.46 for day 1 [baseline parasite density 10^9^–10^10.5^], 2.93 for day 2 [baseline parasite density 10^10.5^–10^12^] and 4.34 for day-3 assessment [> 10^12^]) was applied on the *Ratio*_*i*_. If the *Ratio*_*i*_ value was below the “cut-off” value, the patient was classified to have an artemisinin resistant infection as below.

10 $${\text{Classification}} = \left\{ {\begin{array}{*{20}c} {Resistant, \left( {if \,Ratio_{i} < {\hbox{``}}cut{\text{-}}off{\hbox{''}}} \right)} \\ {Sensitive, \left( {if\, Ratio_{i} > {\hbox{``}}{cut{\text{-}}}off{\hbox{''}}} \right)} \\ \end{array} } \right.$$

Whenever the parasite density value at the proposed assessment day was below LLOQ, the patient was directly classified as having an artemisinin sensitive infection and the ratio calculation was not performed. This classification was compared to patient’s mixture assignment from the model probability estimates, which was regarded as the “truth”. A sensitivity analysis was performed on this comparison and the performance of the nomogram was evaluated.

## Results

A total of 53 patients were recruited to the study and 1 patient was excluded from the study for not meeting the inclusion/exclusion criteria. Two patients were excluded from the pharmacokinetic analysis since they were missing important covariates as well as pharmacokinetic and pharmacodynamic data. Baseline characteristics for the studied population are presented in the Table 1.

**Table 1:** Baseline study demographics

Characteristics	Median (interquartile range)
Weight (kg)	50.0 (46.0–53.5)
Age (years)	25.5 (21.5–39.5)
Oral temperature at enrollment (°C)	38.4 (37.6–39.1)
Haemoglobin (g/dl)	12.4 (10.6–13.7)
Baseline parasite density (parasite/μl)	29,900 (15,200–129,000)
Fever clearance time (day)	3 (2–4)

### Population pharmacokinetics

**Fig. 1:**
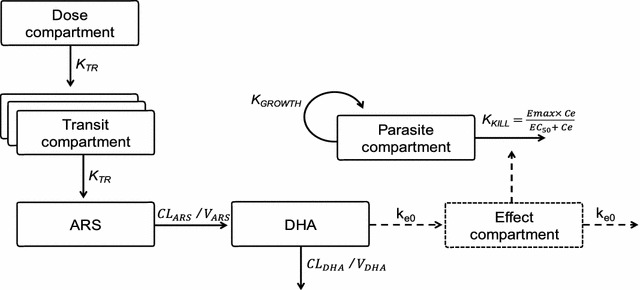
Schematic representation of the final population pharmacokinetic-pharmacodynamic model for parent compound (artesunate; ARS) and its active metabolite (dihydroartemisinin; DHA) in patients with uncomplicated *P. falciparum* malaria. Ce, predicted DHA concentration in the effect compartment; CL, elimination clearance; EC_50_, the DHA concentration which produces 50% of maximum parasite killing effect; Emax, maximum parasite killing effect; k_e0_, effect compartment rate constant governing the delayed drug effect; K_GROWTH_, parasite multiplication rate, fixed to tenfold multiplication per 48-h cycle; K_TR_, first order transit absorption rate constant; V, apparent volume of distribution

**Fig. 2:**
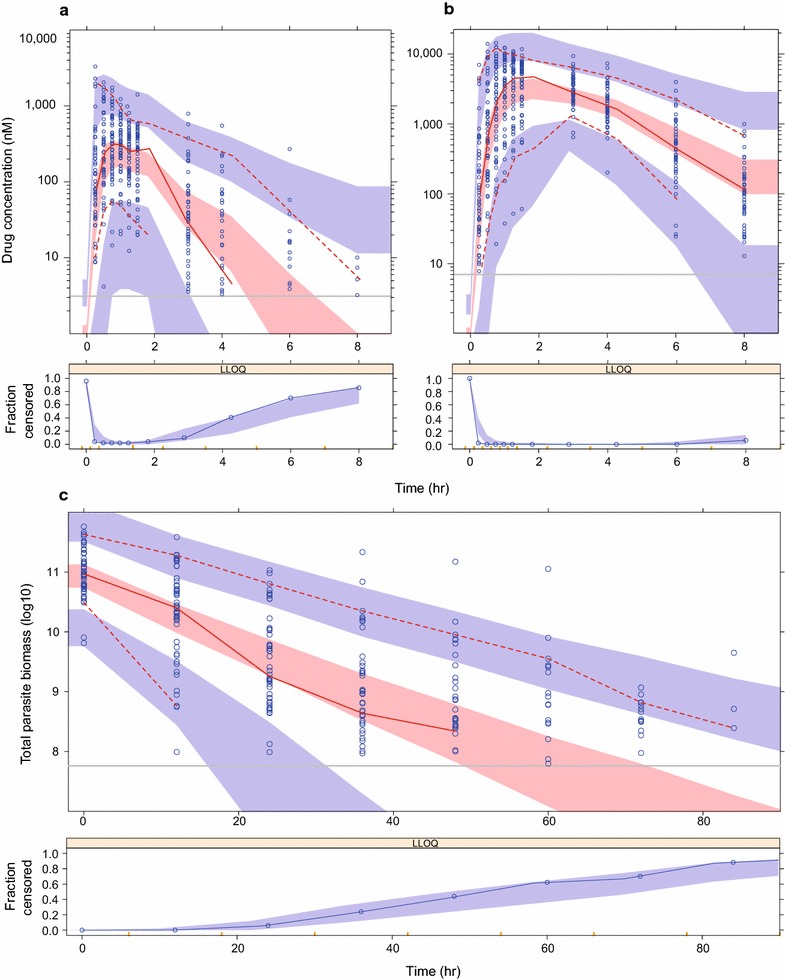
Visual predictive check of final population pharmacokinetic model of artesunate (**a**) and dihydroartemisinin (**b**), and population pharmacodynamic model (**c**) in patients with uncomplicated *P. falciparum* malaria. The open circles are observed data points, the solid red line represents the 50th percentile of observed data; dashed red lines represent the 5th and 95th percentiles (pharmacokinetic model) and the 10th and 90th percentiles (pharmacodynamic model) of observed data; shaded areas are the model predicted 95% confidence intervals of the simulated percentiles; vertical grey lines represent the lower limit of quantification (LLOQ) for artesunate (3.12 nM), dihydroartemisinin (7.02 nM) and parasite density (10^7.73^). The lower panels show the fraction of observed data below the LLOQ (open circles) overlaid with the 95% prediction interval of the fraction of simulated data below the LLOQ (shaded area)

The population pharmacokinetic properties of ARS and DHA were best described using a single disposition compartment model for each of the drug molecules (Fig. 1). The absorption was described by a transit compartment (n = 3) model, which was superior to other absorption models. Allometric scaling of all disposition parameters, centered by the median weight of 50 kg improved the model fit. Malaria disease was implemented a priori as a time-varying covariate on MTT and F, generating a decreased MTT and increased F with increasing parasite counts. No other covariates had a significant impact on the pharmacokinetic parameters in the final model. BSV was maintained in all parameters and the eta shrinkages computed in the final pharmacokinetic model were moderate to low (CL_ARS_ = 36.6%, V_ARS_ = 14.8%, CL_DHA_ = 38.6%, V_DHA_ = 32.4%, MTT = 7.01% and F = 20.2%) while epsilon shrinkages were low (14.1% for ARS and 10.0% for DHA). Simulation-based diagnostics (i.e. VPC) showed satisfactory predictive performance of the final pharmacokinetic model describing ARS and DHA (Fig. 2). Final population pharmacokinetic parameter estimates for ARS and DHA are presented in Table 2.

**Table 2:** Parameter estimates of the final pharmacokinetic-pharmacodynamic model

Parameter	Estimates (% RSE)	95% CI	%CV BSV (% RSE)	95% CI
Pharmacokinetics				
Artesunate				
F (%)	*100 fix*	–	31.2 (29.4)	19.3–50.8
MTT (h)	1.34 (18.8)	1.04–1.96	85.3 (24.9)	65.7–133.0
CL_ARS_/F (l/h)	1750 (8.55)	1570–2090	26.8 (44.3)	11.9–39.1
V_ARS_/F (l)	1300 (12.6)	1110–1660	74.7 (27.3)	57.8–129
RUV (%)	73.2 (3.95)	69.3–78.7	–	–
Dihydroartemisinin				
CL_DHA_/F (l/h)	76.7 (6.99)	69.9–87.8	21.3 (30.3)	13.3–88.1
V_DHA_/F (l)	102.0 (8.95)	89.5–119.0	31.6 (40.5)	21.3–131.0
RUV (%)	58.5 (3.34)	56.6–63.4	–	–
Covariate effects				
^a^PARA_MTT_ (Log10 parasitaemia)	0.115 (8.88)	0.121–0.156	–	–
^a^PARAmax_F_	1.51 (11.9)	1.35–2.02	–	–
^a^PARA50_F_ (Log10 parasitaemia)	8.32 (3.58)	8.19–9.21	–	–
Pharmacodynamics				
K_GROWTH_ (48 h^−1^)	*10 fix*		–	
BASE_PARA_ (Log_10_)	11.0 (0.704)	10.8–11.1	4.4 (19.6)	3.13–5.78
k_e0_ (h^−1^)	0.123 (33.1)	0.0584–0.188	–	
^a^EC_50_ (nM)	30.4 (34.2)	13.5–46.1	–	
^a^Emax_S_ (h^−1^)	0.268 (5.89)	0.242–0.295	^b^49.0 (22.4)	34.3–70.1
Emax_R_ (h^−1^)	0.155 (6.08)	0.142–0.172	12.2 (45.5)	6.54–35.8
P_MIX, resistant_ (%)	56.1 (20.9)	39.1–73.8	–	
RUV (%)	33.3 (5.91)	30.5–37.1	–	

Coefficient of variation (%CV) of between subject variability (BSV) was calculated as 100 × (variance-1)^1/2^. Relative standard errors (% RSE) were calculated as 100 × (standard deviation/mean). The 95% confidence intervals (95% CI) of parameter estimates were obtained with the Sampling Importance Resampling (SIR) approach

*ARS* artesunate, *BASE*_*PARA*_ baseline parasitaemia, *CL* clearance, *DHA* dihydroartemisinin, *F* bioavailability, *K*_*GROWTH*_ parasite multiplication per 48 h parasite cycle, *MTT* mean transit time, *PARA*_*MTT*_ estimated linear effect of parasite density on MTT, *PARAmax*_*F*_ maximum effect of parasite density on F, *PARA50*_*F*_ parasite density which produces 50% of the maximum covariate response, *P*_*MIX, resistant*_ probability of having an artemisinin-resistant infection, *V* volume of distribution, *EC*_*50*_ the DHA concentration which produces 50% of maximum parasite killing effect, *Emax*_*R*_ maximum parasite killing effect of a resistant parasite population, *Emax*_*S*_ maximum parasite killing effect of a sensitive parasite population, *k*_*e0*_ effect compartment rate constant governing the delayed drug effect, *RUV* unexplained residual variability

^a^Estimation of these parameters were obtained by applying a frequentist prior approach using a previously published PK/PD model developed on data from Thailand and Cambodia (37)

^b^BSV (%CV) of Emax_S_ was calculated based on simulations (10,000 patients) with an estimated variance of 0.430 and the applied transformation presented in Eq. 7

### Population pharmacodynamics

The final pharmacokinetic-pharmacodynamic model is illustrated in Fig. 1. A delayed effect model showed a significantly better model fit compared to a direct effect model, and resulted in a half-life of the effect delay of 5.64 h. A mixture model implementation on Emax (to distinguish the parasite clearance between resistant and sensitive parasites), resulted in a better model fit compared to when implementing the mixture on EC_50_, with no additional benefit on having a mixture on both parameters. Sensitive infections were characterized by an Emax estimate of 0.268 h^−1^ (5.89% RSE) compared to 0.155 h^−1^ (6.08% RSE) for the resistant infections, with an estimated 56.1% of patients having a resistant infection. EC_50_ was estimated to 8.64 ng/ml (30.4 nM) but with a relatively high uncertainty (34.2% RSE). There were no significant covariates in the final pharmacodynamic model. The eta shrinkage was 35.0, 18.0 and 27.0% for Emax (sensitive), baseline and Emax (resistant) parameters, respectively, while epsilon shrinkage was 6%. The final pharmacokinetic-pharmacodynamic model parameters and VPCs are presented in Table 2 and Fig. 2.

### Resistance nomogram

Data from the current analysis were used as external validation for a recently developed nomogram from a study in Thailand and Cambodia [4, 36]. Applying the baseline-adapted nomogram to identify patients with resistant parasite infections, resulted in 90.1% overall sensitivity and 92.1% overall accuracy compared to 55.1 and 75.2%, respectively, using the traditional day-3 positivity test. Complete performance of the baseline-adapted nomogram in comparison to the currently used day-3 positivity test is presented in Table 3.

**Table 3:** Predictive performance of the baseline-adapted nomogram and the day-3 positivity test

Statistics metric	Baseline-adapted nomogram		Day-3 positivity test
Negative results (N)	The nomogram predicts the individual parasite density ratio (*Ratio*_*i*_) to be *above* the “Cut-off”		Non-resistant if observed parasitaemia is *below* LLOQ at day 3
Positive results (P)	The nomogram predicts the individual parasite density ratio (*Ratio*_*i*_) to be *below* the “Cut-off”		Resistant if observed parasitaemia is *above* LLOQ at day 3
True positive (TP)	The approach predicts correctly the patient to have a *resistant* infection		
True negative (TN)	The approach predicts correctly the patient to have a *sensitive* infection		
Sensitivity $\frac{TP}{TP + FN}$	Probability of predicting correctly patients with *resistant* infections	90%	55%
Specificity $\frac{TN}{TP + FP}$	Probability of predicting correctly patients with *sensitive* infections	95%	95%
Accuracy $\frac{TP + TN}{TP + TN + FP + FN}$	Proportion of all correct predictions	93%	75%

## Discussion

The spread of artemisinin resistance is threatening the effectiveness of artemisinin-based combination therapies, and it is crucial to monitor the spread and development of artemisinin resistance in Southeast Asia and elsewhere.

The final population pharmacokinetic model of ARS-DHA was similar to that recently developed on data from a study conducted in Thailand and Cambodia [36]. The absorption of ARS was described using a transit compartment absorption model followed by a single distribution compartment for both ARS and DHA. The estimated population pharmacokinetic parameters were in good agreement with that estimated in previous studies, except for V_ARS_, which was somewhat larger in this study [27, 37, 38]. Data presented here cannot elucidate the reason for potential systematic differences but it is well known that ARS is unstable and can rapidly undergo ex vivo conversion to DHA if not collected optimally [16, 39]. Thus, potential differences might be due to slightly different collection procedures in different studies. It was not surprising that body weight had a significant impact on pharmacokinetic parameters, considering that most physiological parameters scale by body weight. In addition, the malaria disease effect on absorption parameters (MTT and F) described in the previous study from Thailand and Cambodia [36] was implemented a priori. The current study featured plasma concentration data from the first administered dose only and the effect of declining parasite densities (i.e. malaria disease effect) on pharmacokinetic parameters could not have been modeled beyond the first dose. Inclusion of this effect based on prior information was incorporated to prevent bias in the drug potency estimate (EC_50_) since the pharmacokinetic model would have otherwise predicted higher plasma concentrations beyond the first dose.

The pharmacokinetic-pharmacodynamic model developed here described the observed parasite data well. A delayed effect model was superior to a direct effect model, and resulted in a half-life of the effect delay of almost 6 h, which is somewhat shorter to the 10 h described previously in Cambodia and Thailand [36]. The estimated delayed effect might reflect a delayed removal of injured and/or dead parasites, which contributes to an apparent sustained killing of parasites beyond the dosing interval. A frequentist prior based on the Thailand–Cambodia study was included to support the estimation of EC_50_ and the artemisinin sensitive Emax value. Prior information was deliberately not applied for other model parameters such as the artemisinin resistant Emax value since resistance could differ both between regions and over time [40]. However, the results indicated rather similar estimates of resistant infections in Myanmar (performed in year 2011; Emax = 0.155, RSE 6.08%) and Thailand–Cambodia (performed in year 2007; Emax = 0.187, RSE 4.88%). This is in line with a recent molecular genotyping study that demonstrated that the artemisinin resistant genotype appears to have spread mostly throughout the region from 2008 to recent years (2014–2015), rather than developed regionally [11].

A total of 56% of patients recruited in this study was estimated to have artemisinin resistant infections, demonstrating a high prevalence of artemisinin resistance in the region. However, the original parasite clearance analysis of these data reported that 19 out of 52 (36.5%) patients in this study had microscopy detectable parasite densities on day 3 (i.e. day-3 positivity test), which is substantially lower than the model-based analyses (5). This is in line with findings from a previous study in Thailand–Cambodia that also suggested that the day-3 positivity test underestimates the number of artemisinin resistant infections compared to a model-based analysis. A likely explanation could be the inability of the day-3 positivity test to take into account the impact of baseline parasite biomass.

The baseline-adapted nomogram was developed based on the relationship that the baseline parasite density is directly proportional to the parasite clearance time, assuming similar drug dependent elimination of parasites [36]. The presented nomogram was suggested to perform better than the commonly used day-3 positivity test, and to have high sensitivity in identifying patients with artemisinin resistant infections [36]. External validation of the nomogram using the data collected in this study in Myanmar showed an overall performance of 90% sensitivity and accuracy, exceeding the expectations of 80% sensitivity and accuracy presented previously [36]. This can be attributed primarily to few patients with low baseline parasite densities (i.e. below 10^10.5^ parasites were the nomogram is known to be less accurate). The reference test of day-3 positivity demonstrated similar inadequate sensitivity (55%) and overall accuracy (75%) as previously concluded. The developed baseline-adapted nomogram offered a high overall accuracy, but primarily a better sensitivity to identify artemisinin resistant malaria infections compared to the traditional day-3 analysis. It also provides a simplified and field-adapted identification of resistant infections by using only one parasite measurement post-dosing. However, the use of the nomogram requires further validation in different epidemiological settings, in adults and children with different levels of immunity as well as different *kelch 13* mutations.

The high prevalence of artemisinin resistance found in this study supports the concern that artemisinin resistance is spreading in the Greater Mekong sub-region. A widespread resistance to artemisinins could potentially reverse the positive trend of recent years of declining morbidity and mortality from malaria [1]. Therefore, it is imperative that containment efforts are scaled-up throughout Myanmar to stop the artemisinin resistance from spreading. In areas where artemisinin resistance is already prevalent, appropriate actions needs to be taken to mitigate the effects, especially the impact on selection for partner drug resistance. Prolonged artemisinin treatment from 3 to 5 days or administration of triple artemisinin-based combinations has been proposed as potential interventions to reduce the pressure on the partner drug and thus combat artemisinin resistance [41, 42].

## Conclusion

The pharmacokinetic-pharmacodynamic model developed here was able to describe the concentration-effect relationship of ARS and DHA in southern Myanmar. The model was able to confirm a high level of artemisinin resistance in this region. Urgent containment efforts and clinical and parasitological monitoring should remain a high priority [43]. In addition, the predictive value of a simple baseline-adapted nomogram for identification of artemisinin-resistant infections was evaluated and outperformed the traditionally used day-3 positivity test and supports its implementation in clinical use.
